# Chemically Synthesized AgNPs and *Piriformospora indica* Synergistically Augment Nutritional Quality in Black Rice

**DOI:** 10.3390/jof9060611

**Published:** 2023-05-25

**Authors:** Shikha Solanki, Samta Gupta, Rupam Kapoor, Ajit Varma

**Affiliations:** 1 Amity Institute of Microbial Technology, Amity University, Uttar Pradesh, Sector-125, Noida 201303, India; solankishikha3012@gmail.com; 2 Department of Botany, University of Delhi, Delhi 110007, India

**Keywords:** nanoparticles, endophytic fungi, mineral nutrients, amino acids, crop productivity, anthocyanin

## Abstract

The use of biofertilizers has been the spotlight of research aiming to mitigate the food security threat as well as to restore the fertility of agricultural lands, for decades. Several studies are being conducted to unravel the role and mechanisms of plant growth-promoting microbes. In the present research, we evaluated the effect of silver nanoparticles (AgNPs) and *Piriformospora indica* on the growth and nutritional enhancement of black rice (*Oryzae sativa.* L.) individually and in combination. Among the different treatment conditions, the AgNPs + *P. indica* treatment led to a significant (*p* ≤ 0.05) increase in morphological and agronomic parameters. In comparison to the control, the percentage increase in plant height in AgNPs-treated black rice was 2.47%, while that for the treatment with only *P. indica* was 13.2% and that for the treatment with both AgNPs + *P. indica* was 30.9%. For the number of productive tillers, the effect of AgNPs in comparison to the control was non-significant; however, the effect of *P. indica* and AgNPs + *P. indica* showed a significant (*p* ≤ 0.05) increase of 13.2% and 30.9% in both the treatments, respectively. Gas chromatography mass spectrophotometry analysis of grains revealed that the contents of phenylalanine, tryptophan, and histidine (aromatic amino acids) were significantly (*p* ≤ 0.05) increased by 75%, 11.1%, and 50%, respectively, in *P. indica*-treated black rice. Nutrient profiling showed that macronutrients such as potassium, calcium, magnesium were found to be increased by 72.8%, 86.4% and 59.2%, respectively, in the treatment with AgNPs + *P. indica* in comparison to the control plants. Additionally, a significant (*p ≤* 0.05) increase of 51.9% in anthocyanin content was observed in AgNPs + *P. indica*-treated black rice. The *P. indica* treatment also showed improved growth and augmented nutrient contents. From this study, we were able to understand that AgNPs + *P. indica* treatment would be a better plant growth-promoting factor and further evaluation would enable us to obtain a clear picture of its mechanisms of action.

## 1. Introduction

Scientists are increasingly in agreement with the fact that human activity, in addition to natural factors, is primarily to blame for the increase in the quantity of greenhouse gases in the atmosphere [1]. There is a multitude of channels via which “global climate change” may impact human prosperity. In addition to the negative impacts of global climatic change, the steady growth in population only exacerbates the existing “Food Security” dilemma. Agrochemicals, which include fertilizers and insecticides, have become an indispensable part of modern agriculture since they are needed for increasing crop yields [2]. There is a disadvantage to the extensive use of chemical fertilizers, notwithstanding their usefulness in boosting crop output and meeting the food demand. According to studies and reports over the past three decades, the usage of these agrochemicals has caused exceptionally high levels of soil and water contamination [3,4,5]. Knowledge of the close association between soil microbiota and plants and their synergic mutuality, that represents a positive co-relation, has been crucial to various alternative processes to evolve sustainability in the agriculture sector to decrease or even recover the use of pesticides and fertilizers [6].

The relationship between plants and fungus is incredibly prevalent. This relationship between plants and endophytes has endured for around 400 million years, according to fossil evidence [7]. The direct benefits of interacting with endophytic fungus include an increase in nutrient uptake and phytohormone synthesis, which is associated with an increase in the production of biomass, root system expansion, plant height increase, reproduction weight increase, and yield increase. Due to these advantages, endophytes are known as biofertilizers [8]. *P. indica*, an endophytic fungus, belongs to the Serendipitaceae family and has been studied for its effect on plant growth and nutrient acquisition in different crops such as wheat, rice, maize, etc. [9]. In recent years, nanotechnology has drawn greater attention in the agriculture industry than traditional agricultural practices have [10]. Nano-bioformulations, plant growth promoters and regulators, nanofertilizers, nutrition management, and phytopathogen protection are among the numerous agricultural applications of nanoparticles [11]. Several studies have documented the beneficial effects of nanoparticles (NPs) on various crops such as maize, rice, wheat, etc. [12,13,14,15]. There are concerns regarding the extensive application of AgNPs, which might result in high environmental release, causing pernicious effects on the aquatic organisms that undergo constant exposure to them [16]. However, using fungi as stabilizing agents in the synthesis of AgNPs via biogenic pathways is good due to the low toxicity of the residues. Combining fungus with AgNPs forms a biomolecule coating on the fungus-derived NPs, which can enhance stability and biological activity. Studies have reported that AgNPs in association with fungi have low toxicity and good biocompatibility [17]. Several studies have documented the beneficial effects of AgNPs on various crops such as *Triticum aestivum, Brassica juncea* and *Vaspertilio sinensis* under greenhouse conditions [18]. The germination rates of three plant species, corn, watermelon, and zucchini, were also augmented in response to AgNPs as examined by [19]. AgNPs help to improve crop yield in agriculture by enhancing the germination of seeds and growth of plants, and the response of plants to AgNPs is dependent on the dosage. While certain AgNPs concentrations can promote the growth of plants in comparison to that of un-exposed plants, higher concentrations may have a repressive effect on the growth of plants [20,21]. Although AgNPs have been reported to cause growth retardation in plants [22,23], when combined with *P. indica*, they showed positive growth patterns as observed in our previous study [24]. Therefore, the conjugation of these nanoparticles with endophytic fungus could have a strong effect on crop productivity.

Rice, being the pivotal nutrient source for almost the entire global population, has always been a spotlight of research for more than decades [25]. In Asia, black rice (*Oryza sativa* L.), has been cultivated for ages [26]. Black rice contains higher levels of nutrients (Fe, P, Ca, and Zn) and dietary fiber than white and brown rice do. The physiological and pharmacological potential of black rice has been well demonstrated by research [27]. In addition, lipids, amino acids, vitamins (A, B complex, and E), mineral nutrients (Fe, K, Zn, Mg, Cu, P, and Mn), dietary fiber, phenolic compounds, anthocyanins, and tocopherols are available in the embryo and bran layers of black rice [28]. Due to its nutrient-dense composition, its consumption was restricted to privileged groups, and it was hence denoted “Imperial Rice” [29]. There are 200 different types of black rice around the globe. China accounts for 62% of the global production of black rice. Several ways have been employed throughout the years to increase the yield of black rice to meet the nutritional needs of the general population [30]. Endophytes can affect rice growth via phytohormone synthesis such as that of auxins (IAA) and gibberellins (GAs), the solubilization of inorganic phosphate, nitrogen fixation, and the inhibition of ethylene levels via the synthesis of ACC Deaminase. Whether or not or how these growth-promoting microorganisms interact actively with rice is not understood [31]. Endophytes of *P. glomerata*, and *P. formosus* fungi that produce IAA and GA also significantly increase rice chlorophyll content, shoot length, and biomass [32]. Similarly, the utilization of NPs to enhance rice growth has also reached several milestones. The use of AgNPs significantly promoted the growth of rice seedlings in terms of root length, shoot length, fresh weight, and dry weight [33]. A similar study on rice was performed to enhance germination efficiency, root length, shoot length and leaf length using zinc nanoparticles (ZnONP) [34]. The molecular mechanism behind growth enhancement when using NPs on rice was elucidated by [35]. The effect of AgNPs on rice germination was attributed to the enhanced water absorption potential caused by the upregulation of aquaporins (PIP1 and PIP2). Very few studies have elucidated the combinatorial role of NPs and endophytic fungi on crop improvement [24,36,37,38]. These studies demonstrate the potential benefits of combining nanoparticles and endophytic fungi for crop improvement. However, more research is needed to explicate the mechanisms underlying these effects and to optimize the application of these treatments in agricultural practices. The present study evaluates the potential of the synergistic effect of AgNPs and *P. indica* treatments on the agronomic and morphological parameters and nutritional enhancement of black rice.

## 2. Materials and Methods

### 2.1. Synthesis and Characterization of Silver Nanoparticles

AgNPs were synthesized via a temperature-dependent chemical reduction method by slightly modifying the methodology of [39] at SCNS, JNU.

**Figure 1:**
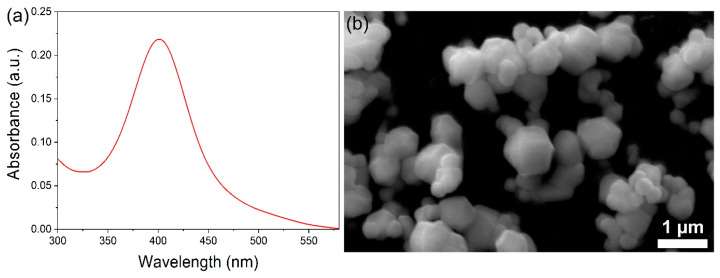
(**a**) UV-Visible spectroscopy peak of AgNPs; (**b**) scanning electron micrograph of AgNPs.

The characterization of AgNPs was carried out using a UV-visible spectrophotometer and scanning electron microscopy (SEM) [40,41]. UV-visible absorption spectrophotometry revealed a peak at about a wavelength of 400 nm, which is typical of a well-dispersed AgNP SPR peak (Figure 1a). Utilizing SEM, the prepared AgNPs were examined. The size of AgNPs ranged from 50 to 150 nm on average, and they were hexagonal in shape (Figure 1b).

### 2.2. Experimental Setup

The black rice variety Chakhao Poireiton utilized for the present study was obtained from Manipur, North-East, India. The seeds were rinsed with DW to remove any surface debris followed by surface sterilization in a 3% sodium hypochlorite solution for 5 min with intermittent stirring, then washing with DW [42].

**Figure 2:**
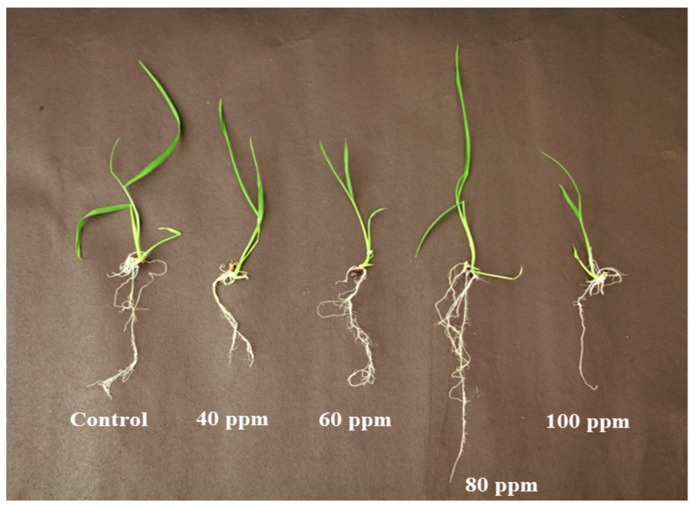
AgNPs optimization in black rice grown in modified Morishige and Skoog medium containing different concentrations of AgNPs.

In vitro germination of black rice seeds was performed as shown in Figure 2 for the optimization of the AgNPs concentration in black rice, where five different AgNPs concentrations were used: control (untreated), 40 ppm, 60 ppm, 80 ppm, and 100 ppm. Maximum growth was observed at 80 ppm, as observed by the increased root and shoot length after 12 days at 28 °C.

*P. indica*, a rhizospheric endophytic fungus, preserved at Amity Institute of Microbial Technology (AIMT) under the reference number DSM 11827, was used in the present study. The uncontaminated inoculum was procured from the AIMT lab and was sustained in a 4% jaggery medium, using the procedure detailed in Singhal et al.’s study (2017) [43]. The selection of the concentration of 5 *×* 10^5^ spores/mL^−1^ of *P. indica* for the present research experiments was made based on the findings of an earlier study conducted by Dabral et al. (2019) [44].

A pot experiment was conducted at the greenhouse of AIMT, wherein a modified Morishige and Skoog medium was employed for the in vitro germination of black rice seeds. The seedlings were transferred to larger earthen pots measuring 25 cm in diameter, which contained a mixture of sand, vermiculite, and sterile soil in a 1:1:1 ratio, after being hardened for 21 days. The experiment comprised four treatments, including untreated black rice (control), black rice treated solely with AgNPs at 80 ppm, black rice treated solely with *P. indica* at 5 × 10^5^ spores/mL, and black rice treated with AgNPs + *P. indica* at 5 × 10^5^ spores/mL. There were three replicates of each treatment, totaling twelve pots. Initially, five seedlings were planted per pot of each treatment, which were later thinned to four plants in each pot, making it twelve plants per treatment. The plants were grown and observed for eight months under controlled greenhouse conditions with a relative humidity of 85% and a temperature of 28 °C. No additional fertilizer was provided during the growth period. While the agronomic and morphological parameters were evaluated after 7 months (early grain stage), for the analysis of the nutritional value of grains, eight-month-old plants (late grain stage) were used.

### 2.3. Morphological and Agronomic Characterization

To study the performance of black rice under different treatment conditions, morphological and agronomic characters such as plant height, number of productive tillers, flag leaf length, panicle length, single plant yield, and 100-grain weight were recorded for the control and treated plants. The seeds harvested from the different treatment conditions were then utilized for the assessment of total protein content, total fat content, anthocyanin content, nutrient profiling and amino acid profiling.

### 2.4. Total Protein Content

The Kjeldahl technique as reported by [45] was used to assess the total protein content of seeds grown under different treatment conditions. Powdered rice grains (0.25 g) were placed in digestion tubes, and 3 mL of concentrated H_2_SO_4_ was added to oxidize the organic substance and release the reduced nitrogen as (NH_4_)_2_SO_4_. A combination of potassium sulphate, copper sulphate, and titanium dioxide was added to the aforementioned solution in order to boost the boiling point and function as a catalyst to accelerate the reaction. After adding all these chemicals, samples were left to digest for two hours until the dark-colored solution became colorless. NaOH (45%) was used to conduct further distillation, which converted ammonium sulphate to ammonia, showing the sample’s nitrogen content. The end of the condenser of the distillation unit was dipped in a boric acid solution. The sample’s ammonia reacted with boric acid and changed the solution’s color from purple to green; a sample volume of roughly 50 mL was taken and backtitrated with 0.05 N HCl until the green color turned pale pink. The blank value was used to calculate the quantity of nitrogen in the sample using the titer value. The protein content was expressed in g/100 g of grains.

### 2.5. Total Fat Content

The total fat content present in the rice samples was estimated using Soxhlet method following the protocol of [46]. Around 30 g of the grain sample was ground with a mortar and pestle. Two grams of the sample was added into the thimble. An amount of 350 mL of petroleum ether was taken into the flask and glass beads were added to it. The flask was then kept in the Soxhlet extractor for at least six hours. After extraction and prior to drying, thimble samples were removed from the Soxhlet apparatus and allowed to air dry in a hood overnight, followed by drying in a vacuum oven at 70 °C, for 24 h. The samples were then cooled in a desiccator and reweighed. The fat content was expressed in g/100 g of grains.

### 2.6. Anthocyanin Content

The anthocyanin content present in the rice seeds of different treatment conditions was determined via LCMS/MS analysis following the methodology of [47]. One gram of rice grains was ground well and added to 10 mL of a solvent containing methanol, H_2_O and formic acid in a ratio of 70:28:2 (*v*/*v*). The samples were then mixed well for 10 min. After complete blending, the samples were filtered through a 0.45 µm filter. The samples were then subjected to LCMS/MS analysis using 6470 Triple Quadrupole LC/MS, Agilent, USA. The column used for the analysis was Poroshell 120 SB-C18, 4.6 mm × 75 mm, 2.7 µm. An electrospray ionization (ESI) interface in the positive ionization mode was used. The capillary voltage was set to 4 kV, the cone voltage was set to 40 V, the desolvation temperature was set to 350 °C, and the desolvation gas flow was set to 600 L/h. The data were analyzed using MassLynx software, and further used to identify and quantify the anthocyanin content in the sample by comparing them to the standard (Cyanidin 3-glucoside).

### 2.7. Nutrients Profiling

For nutrient profiling, sample preparation was conducted using a microwave-assisted digestion protocol explained in [48]. Around 0.5–1.0 g of sample from each treatment condition was taken in the digestion tube and 3 mL of milliQ water was added and mixed well. To this, 8 mL of nitric acid and 1 mL of HCl were added and incubated for 30–45 min. After digestion, the sample volumes reached 50 mL and elemental analysis was performed in iCAP 7000 Series ICP-OES, Thermo Scientific (Waltham, MA, USA).

### 2.8. Amino Acids Profiling

Amino acids in rice grains were quantified per the methodology described by [49]. One gram of the rice seeds was powdered, added to a tube containing 80% (*v*/*v*) ethanol (5 mL) and vortexed vigorously for 5 min. The samples were then centrifuged (5000× *g* rpm; 10 min; 4 °C) and the supernatant obtained was filtered through a millipore filter (0.45 µm pore size). The extraction procedure was performed twice, and the resulting extracts were dried on a rotary evaporator, after which the dried residue was collected and dissolved in water (1 mL) for further derivatization and HPLC analysis performed in Agilent 1200 Series Gradient HPLC System, Agilent, Santa Clara, CA, USA.

### 2.9. Statistical Analysis

The data analysis was conducted using the Statistical Package for the Social Sciences Statistics software version 21.0 (SPSS Inc., IBM Corporation). To compare the individual mean, a one-way analysis of variance (ANOVA) was performed, and the differences were assessed using Tukey’s honestly significant difference (HSD) post hoc test with the significance level set at *p ≤* 0.05. The mean values of three biological replicates were presented along with their standard deviations (SD).

## 3. Results

### 3.1. Effect of AgNPs on P. indica

**Figure 3:**
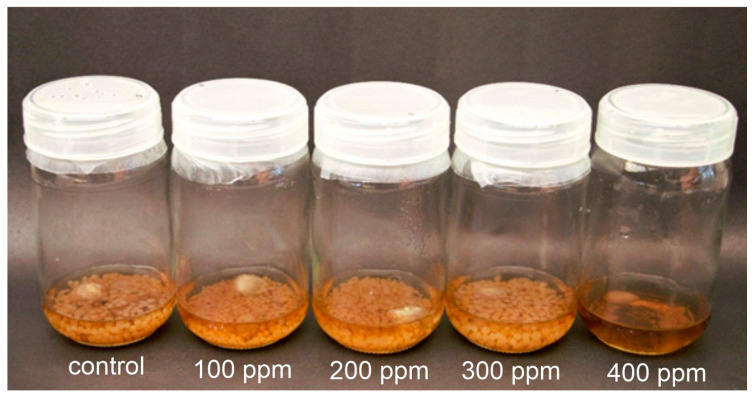
The growth enhancement of *P. indica* when treated with AgNPs in 4% jaggery broth media prepared at pH 6.7; left to right: control, 100 ppm, 200 ppm, 300 ppm, and 400 ppm.

Fungal spores treated with AgNPs at 300 ppm in a broth medium (consisting of a 4% jaggery culture medium in 10 mL of distilled water) exhibited an increase in fungal mycelial growth, (Figure 3) as well as in spore number, and spore size after 10 days of incubation. The visible enhancement of *P. indica* growth was dependent on the concentration of AgNPs, with the size and biomass of the colonies progressively increasing from 100 ppm to 300 ppm and eventually retarding at 400 ppm.

### 3.2. Morphological and Agronomic Characters

Among the morphological and agronomic parameters, the plant height, panicle length, yield per plant and 1000-grain weight were significantly (*p ≤* 0.05) increased in all the treatments compared to those of the control; however, the effect of the treatments was non-significant in terms of the number of productive tillers and flag leaf length (Table 1).

In comparison to the control, the percentage increase in plant height in AgNPs-treated black rice was 2.47%, that for the treatment with only *P. indica* was 13.2% and that for the treatment with both AgNPs + *P. indica* was 30.9%. For the number of productive tillers, the effect of AgNPs in comparison to that in the control was non-significant; however, the effect of *P. indica* and AgNPs + *P. indica* showed a significant (*p ≤* 0.05) increase of 18.1% and 45.4%, respectively.

**Figure 4:**
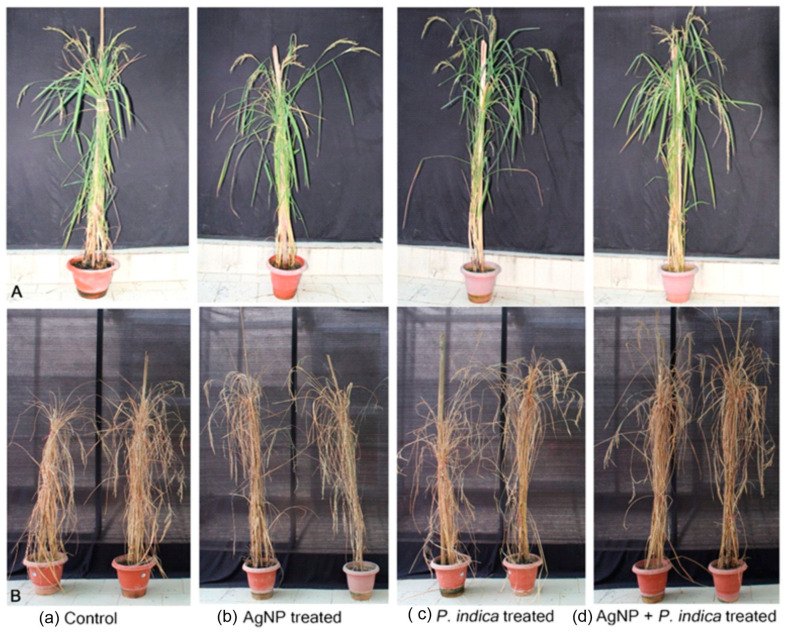
Pot culture experiment of black rice *(Oryza sativa* L.) (**A**) Early grain stage and (**B**) mature grain stage. (**a**) Control, (**b**) only AgNP-treated, (**c**) only *P. indica*-treated (**d**) AgNP +*P. indica*-treated.

The panicle length also increased by 47.8% in AgNPs + *P. indica*-treated black rice compared to that of the control. The only AgNPs-treated black rice showed a 5.1% increase and the only *P. indica*-treated black rice showed a 30.7% increase in comparison to that of the control. The yield per plant showed a significant (*p ≤* 0.05) increase of 73.1% in the treatment with both AgNPs + *P. indica*, of 61.7% in the treatment with only *P. indica* and of 27.4% in the treatment with only AgNPs- in comparison to that of the control (Figure 4).

**Figure 5:**
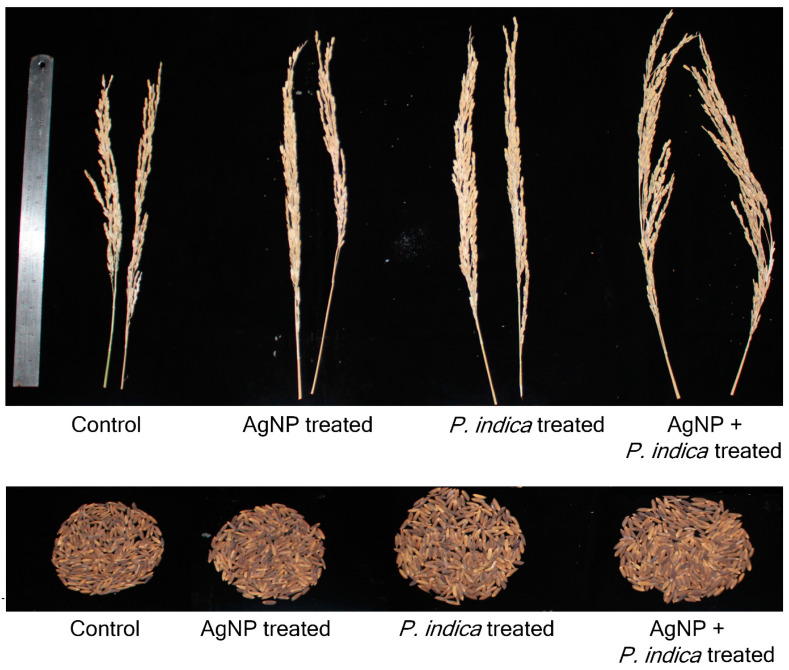
Panicle length and yield of black rice in control, AgNPs treated, *P. indica* treated, AgNPs + *P. indica*-treated, respectively.

The positive effect of the AgNPs + *P. indica* treatment was also highest for the weight of 1000 grains as evidenced by a significant (*p ≤* 0.05) increase of 65.1% compared to that of the control plants. The effect of the treatment with only AgNPs on black rice plant height was a 2.4% increase, that on the flag leaf length was a 1.8% increase, that on the panicle length was a 5.1% increase and that on the 1000-grain weight was a 20.9% increase, whereas the treatment of black rice with *P. indica* alone showed an increase of 46.5% of the 1000-grain weight (Figure 5).

### 3.3. Total Protein, Fat and Anthocyanin Content

One-way analysis of variance showed that the effect of treatments was non-significant on the contents of total protein and fat (Table 2). However, a significant (*p ≤* 0.05) increase in the content of anthocyanin was made evident by the increased values of 51.9% in the AgNPs + *P. indica*-treated black rice in comparison to that of the control. The other treatment conditions—that is, the treatments with only AgNPs and *P. indica* alone—showed non-significant results with a 2.5% increase in anthocyanin content compared to that in the control plants.

### 3.4. Nutrients Profiling

Among the micronutrients, the values of Zn and Co were significantly increased (*p ≤* 0.05) by 8.7% and 9%, respectively, in AgNPs-treated black rice compared to those of the control. However, the effect of AgNPs on the Fe and Mn contents was non-significant. Amongst the macronutrients, only K showed a significant increase (*p ≤* 0.05) of 4.3% in the AgNPs treatment compared to that in the control, whereas the AgNPs effect was non-significant for Mg and Ca contents. The effect of *P. indica* alone was significant (*p ≤* 0.05) for micronutrients Zn, Mo, and Co with an increase of 10.9%, 25% and 18.1%, respectively, compared to those in the control. However, the effect of *P. indica* on Fe, Mn, and Cu was non-significant. The content of the micronutrients, Fe, Mn, Zn, and Mo, were found to be significantly (*p ≤* 0.05) higher in the AgNPs + *P. indica*-treated plants, increasing by 33.3%, 61.1%, 23%, and 52.8%, respectively, when compared to those in the control.

Among the macronutrients, the effect of *P. indica* alone was significant (*p ≤* 0.05) on Mg and K as they were increased by 35.3% and 6.6%, respectively. However, Na showed non-significant results for the treatments with *P. indica* alone. Nutrient profiling also showed that among the macronutrients, the contents of K, Ca, Na, and Mg, were significantly (*p ≤* 0.05) increased by 72.8%, 86.4%, 71.4% and 59.2% in the AgNPs + *P. indica-* treatment compared to those in the control plants (Table 3).

### 3.5. Amino Acids Profiling

Among the analyzed amino acid, the contents of aromatic amino acids such as phenylalanine, tryptophan, and histidine were significantly (*p ≤* 0.05) increased by 75%, 11.1%, and 50%, respectively, in the *P. indica*-treated black rice in comparison to those in the control. The content of aspartic acid, histidine, arginine, tryptophan, leucine, and phenylalanine was significant higher in all the treatments compared to that in the control plants. Proline and glycine showed a significant (*p ≤* 0.05) increase of 66.6% and 50% for the AgNPs + *P. indica*-treated black rice compared to those in the control; however, the values were non-significant for the other two treatments. Methionine also showed a significant (*p ≤* 0.05) increase in the treatment with *P. indica* alone and AgNPs + *P. indica*-treated black rice compared to that in the control. The effect of only *P. indica*-treated black rice in comparison to that in the control was significant (*p ≤* 0.05) with an increase of 33.3% for tyrosine, 50% for valine, 66.6% for isoleucine and 50% for leucine. Phenylalanine and leucine showed a significant increase (*p ≤* 0.05) of 25% and 16.6% in the AgNPs treatment compared to those in the control plants. The values of cysteine increased significantly (*p ≤* 0.05) by 25% and those of arginine increased by 60% for the *P. indica*-treated black rice in comparison to those in the control. The values of glycine, histidine, threonine, tyrosine and methionone showed a negligible change in the only AgNPs-treated black rice (Table 4).

## 4. Discussion

The utilization of NPs for enhanced plant growth has been extensively studied in recent decades in a wide range of crop species (Theusombat et al., 2016; Pestovsky et al., 2017) [50,51]. In rice, the effect of different types of NPs has been studied by researchers across the globe [50,52]. Among the other available NPs, AgNPs are widely used NPs. In the present study, AgNPs were utilized to study their effect on agronomic properties and nutritional properties in black rice. In addition, the effect of the endophytic fungus *P. indica* and the combined effects of *P. indica* along with AgNPs were also evaluated. The black rice seeds treated with AgNPs, *P. indica* and AgNPs + *P. indica* were grown and observed until maturity under greenhouse conditions. The morphological parameters such as plant height, weight of 1000 grains, and panicle length, showed a significant increase (*p* ≤ 0.05) in the *P. indica*-treated plants. However, AgNPs-treated plants showed only a meager difference. AgNPs-treated seeds of jasmine rice, KDML105, showed an enhanced germination rate. It has been documented that AgNP treatment (5 ppm and 100 ppm) improves seed germination in jasmine rice by influencing ROS levels and aquaporins [53]. In our study, we standardized 80 ppm as the optimal concentration and observed a significant increase (*p* ≤ 0.05) in the agronomic parameters: plant height, number of productive tillers, panicle length, yield per plant and 1000-grain weight when combined with *P. indica* in pot culture experiments. The morphological and agronomic parameters play a vital role in sustainable agricultural development as a high yield potential due to *P. indica* helps to ensure food security by increasing crop production which is crucial in a world where the food demand is high.

The effect of AgNPs alone on the number of productive tillers was not significant, whereas a significant growth (*p* ≤ 0.05) enhancement in terms of the number of productive tillers, panicle length, flag length and biomass was observed in black rice treated with *P. indica* alone and with AgNPs + *P. indica*. This shows the positive functional and essential role of *P. indica* and its association with black rice and AgNPs, which has further agricultural implications. In a study conducted by [54], rice cultivar Tainung 67 treated with *P. indica* showed an increased yield as indicated by increasing the panicle number. Further characterization revealed enhanced water stress tolerance via the regulation of stomatal closure and better oxidative stress management. A very limited number of studies have assessed the combinatorial effect of NPs and endophytes. A study conducted on maize growth enhancement via calcium phosphate NPs in conjugation with *P. indica* showed increased chlorophyll content and a significant increase in plant height, root length, and biomass [55]. ZnO nanorod-embedded *P. indica* treatment of *Brassica oleracea* (Broccoli) also showed a significant increase in the germination rate, root length and shoot length [56].

In our study, we observed that AgNP treatment along with *P. indica* led to an increase in the nutritional quality of black rice. Nutrient profiling of the harvested seeds showed an increase in nutrients such as Fe, Mn, Ca, Zn, and Mo in AgNPs + *P. indica*-treated black rice plants. Micronutrients are essential nutrients that are important for growth and development, energy production, immune function, and mental health (Calder et al., 2020) [57]. Zn is essential for immune function. It is necessary for the proper functioning of the immune system. It plays a key role in the development and activation of immune cells and helps protect against infections. Zn deficiency can impair immune function and increase the risk of infections [57]. Iron and Zn are crucial for various physiological functions in the body and increased under AgNPs + *P. indica* treatment. Co is an essential trace element that is required to produce vitamin B12, for energy production, for iron metabolism and for proper brain function [58]. It was also enhanced in the AgNPs + *P. indica* treatment (Table 3).

Potassium, calcium, and magnesium are vital macronutrients for sustaining good health. Potassium regulates fluid balance, maintains blood pressure, supports muscle function, and facilitates nerve transmission as an electrolyte. Consuming adequate amounts of potassium via a balanced diet that includes fruits, vegetables, and whole grains has been demonstrated to decrease the risk of high blood pressure, kidney disease, and stroke [59]. Calcium is essential for healthy bones and teeth, as well as muscle function, nerve signaling, and blood clotting. Consuming enough calcium through a balanced diet can lower the risk of osteoporosis and other bone disorders [60]. Magnesium is involved in various physiological processes in the body, including energy metabolism, protein synthesis, and muscle and nerve function, and is necessary for maintaining a normal heart rhythm and blood pressure. Consuming enough magnesium through a balanced diet that includes nuts, seeds, legumes, and whole grains can lower the risk of heart disease, stroke, and type 2 diabetes [61]. Nutrient profiling resulted in the enhancement of these macronutrients in the AgNPs + *P. indica*-treated black rice in the present study.

Anthocyanins are important bioactive compounds found in many fruits and vegetables that have been associated with various health benefits. Anthocyanins possess potent antioxidant and anti-inflammatory properties and have been linked to enormous health benefits. Anthocyanins may help to decrease the risk of various diseases, such as cardiac, cancer, diabetes, etc. [62]. Anthocyanins can protect against oxidative stress and brain inflammation, and may help to mitigate various neurological problems, such as Alzheimer’s and Parkinson’s disease. [63]. Findings of the study suggest that the total anthocyanin content in AgNPs + *P. indica*-treated black rice was significantly increased in comparison to that in the control, suggesting the better bioactive status of AgNPs + *P. indica*-treated rice compared to that of the control plants. The findings of the present study support the potential of a synergistic effect of AgNPs + *P. indica* treatment of improving agronomic parameters and the nutritional status of black rice. However, to fully elucidate the exact mechanism of such enhancement, further studies are needed.

Aromatic amino acids play a vital role in various physiological processes in the human body. They are essential components of protein synthesis and immune function, and they also have antioxidant activity which can help to protect against oxidative stress [64]. In our research, aromatic acids were enhanced in both *P. indica*- as well as AgNPs + *P. indica*-treated black rice; therefore, ensuring adequate intake of these amino acids in diet is essential to maintain optimal health. Tryptophan deficiency can lead to a decrease in serotonin synthesis [65]. In the current study, tryptophan was enhanced in the AgNPs + *P. indica* treatment, and it helps in the regulation of neurotransmitter cognition. Histidine is involved in protein synthesis, pH regulation, and blood cell maintenance, and it may also have cognitive benefits [66]. A steady increase in histidine in the AgNPs +*P. indica* treatment indicated its importance for maintaining optimal health. Research has shown that aspartic acid is essential for maintaining good health. Aspartic acid supplementation can improve athletic performance by increasing endurance and reducing fatigue [67]. It was enhanced in both *P. indica*- and AgNPs + *P. indica*-treated black rice in our research study. Leucine, proline, and glycine are all important amino acids that play different roles in the human body. Leucine is essential for muscle protein synthesis and the maintenance of muscle mass, proline is important for the formation of collagen and the prevention of protein misfolding diseases, and glycine is involved in the synthesis of important molecules and the regulation of inflammation [68,69,70]. Three of these elements were enhanced in the AgNPs + *P. indica* treatment (Table 4). Therefore, an improvement of these amino acid levels in black rice grain can help to provide a more complete source of protein and nutrition as they help to boost energy metabolism and stimulate protein synthesis by increasing nutritional quality.

## 5. Conclusions

The current study was aimed at elucidating the beneficial effects of AgNPs and *P. indica* on the growth and nutritional acquisition of black rice. The results clearly suggest that *P. indica* treatment in conjugation with AgNPs significantly increased the growth and nutritional content. Further characterization would greatly help in understanding the mechanism which was regulated by the *P. indica* treatment. The association of fungal endophytes and AgNPs, in correlation with the enhancement in its yield, nutritional value addition and crop productivity in black rice has been described. The main aim of our research was to form a unique nanotechnology-integrated fungal endosymbiont that could be used as a “Bio-formulation” in the fortification of crops for sustainability in agriculture.

## Figures and Tables

**Table 1:** Morphological and agronomic parameters of black rice under different treatment conditions.

S. No	Parameters	Control	AgNP Treated	*P. indica* Treated	AgNP + *P. indica* Treated
1	Plant hHeight (cm)	185.9 ± 0.07 ^d^	190.5 ± 0.04 ^c^	210.5 ± 0.01 ^b^	243.5 ± 0.34 ^a^
2	No. of productive tillers	11 ± 0.03 ^c^	11 ± 0.07 ^c^	13 ± 0.03 ^b^	16 ± 0.021 ^a^
3	Flag leaf length (cm)	73.1 ± 0.15 ^c^	74.4 ± 0.05 ^a^	73.4 ± 0.003 ^b^	73.1 ± 0.34 ^c^
4	Panicle length (cm)	23.4 ± 0.19 ^d^	24.6 ± 0.10 ^c^	30.6 ± 0.04 ^b^	34.6 ± 0.50 ^a^
5	Yield per plant (gm)	17.5 ± 0.34 ^d^	22.3 ± 0.01 ^c^	28.3 ± 0.42 ^b^	30.3 ± 0.25 ^a^
6	1000 grain weight (gm)	4.3 ± 0.021 ^d^	5.1 ± 0.006 ^c^	6.3 ± 0.008 ^b^	7.1 ± 0.014 ^a^

Values represent means of three replicates ± SD. Within each row, means followed by same letter do not differ significantly at *p ≤* 0.05, derived from Tukey’s HSD.

**Table 2:** Total protein, fat and anthocyanin content observed in the black rice grains of different treatment conditions expressed in gm/100 gm of seeds.

S. No	Compound	Control	AgNPs Treated	*P. indica* Treated	AgNPs + *P. indica* Treated
1	Total Protein	7.4 ± 0.07 ^a^	7.4 ± 0.13 ^a^	7.5 ± 0.08 ^a^	7.5 ± 0.12 ^a^
2	Total Fat	2.6 ± 0.03 ^a^	2.6 ± 0.04 ^a^	2.6 ± 0.03 ^a^	2.6 ± 0.05 ^a^
3	Anthocyanin	7.7 ± 0.04 ^b^	7.9 ± 0.09 ^b^	7.8 ± 0.07 ^b^	11.7 ± 0.11 ^a^

Values represent means of three replicates ± SD. Within each row, means followed by same letter do not differ significantly at *p ≤* 0.05, derived from Tukey’s HSD.

**Table 3:** Nutrient profiling in the black rice grains of different treatment conditions expressed in mg/100 gm of seeds.

S. No	Compound	Control (mg/100 gm)	AgNPs Treated (mg/100 gm)	*P. indica* Treated (mg/100 gm)	AgNPs + *P. indica*- Treated (mg/100 gm)
1	Iron (Fe)	6.3 ± 0.11 ^b^	6.9 ± 0.11 ^b^	7.3 ± 0.15 ^b^	8.4 ± 0.28 ^a^
2	Manganese (Mn)	3.7 ± 0.24 ^b^	3.7 ± 0.24 ^b^	3.9 ± 0.26 ^b^	5.8 ± 0.64 ^a^
3	Copper (Cu)	0.3 ± 0.02 ^c^	0.2 ± 0.007 ^c^	0.4 ± 0.03 ^b^	0.6 ± 0.04 ^a^
4	Zinc (Zn)	9.1 ± 0.13 ^b^	9.9 ± 0.13 ^ab^	10.1 ± 0.13 ^ab^	11.3 ± 0.10 ^a^
5	Molybdenum (Mo)	3.6 ± 0.34 ^c^	3.5 ± 0.32 ^c^	4.5 ± 0.42 ^b^	5.5 ± 0.25 ^a^
6	Nickel (Ni)	0.23 ± 0.01 ^b^	0.24 ± 0.01 ^b^	0.24 ± 0.01 ^b^	0.38 ± 0.01 ^a^
7	Sodium (Na)	4.3 ± 0.19 ^b^	4.7 ± 0.21 ^b^	4.6 ± 0.21 ^b^	7.2 ± 0.14 ^a^
8	Cobalt (Co)	1.1 ± 0.005 ^d^	1.2 ± 0.006 ^c^	1.3 ± 0.007 ^b^	2.1 ± 0.021 ^a^
9	Magnesium (Mg)	143.7 ± 2.18 ^c^	144.8 ± 2.19 ^c^	194.6 ± 2.95 ^b^	228.9 ± 2.27 ^a^
10	Potassium (K)	267.8 ± 4.10 ^c^	279.4 ± 4.28 ^b^	285.7 ± 4.38 ^b^	462.8 ± 7.05 ^a^
11	Calcium (Ca)	33.3 ± 0.50 ^c^	33.4 ± 0.50 ^c^	54.2 ± 0.82 ^b^	61.9 ± 1.88 ^a^

Values represent means of three replicates ± SD. Within each row, means followed by same letter do not differ significantly at *p ≤* 0.05, derived from Tukey’s HSD.

**Table 4:** Amino acid profiling in the black rice grains of different treatment conditions expressed in gm/100 gm of seeds.

S. No	Compound	Control	AgNP Treated	*P. indica* Treated	AgNP + *P. indica* Treated
1	Aspartic Acid	0.7 ± 0.008 ^d^	0.9 ± 0.01 ^c^	1.2 ± 0.02 ^b^	2.03 ± 0.04 ^a^
2	Serine	0.3 ± 0.01 ^c^	0.3 ± 0.007 ^c^	0.4 ± 0.009 ^b^	0.8 ± 0.01 ^a^
3	Glutamic Acid	15 ± 0.01 ^c^	1.6 ± 0.01 ^c^	3.4 ± 0.03 ^b^	4.1 ± 0.04 ^a^
4	Glycine	0.4 ± 0.02 ^c^	0.4 ± 0.008 ^b^	0.5 ± 0.01 ^a^	0.6 ± 0.01 ^a^
5	Histidine	0.2 ± 0.003 ^d^	0.2 ± 0.008 ^c^	0.3 ± 0.01 ^b^	0.5 ± 0.02 ^a^
6	Arginine	0.6 ± 0.03 ^d^	0.6 ± 0.006 ^c^	0.8 ± 0.008 ^b^	1.4 ± 0.01 ^a^
7	Threonine	0.3 ± 0.011 ^c^	0.3 ± 0.007 ^b^	0.3 ± 0.006 ^b^	0.6 ± 0.01 ^a^
8	Alanine	0.4 ± 0.004 ^c^	0.6 ± 0.05 ^c^	1.0 ± 0.09 ^b^	1.3 ± 0.12 ^a^
9	Proline	0.3 ± 0.007 ^c^	0.4 ± 0.004 ^b^	0.5 ± 0.005 ^a^	0.5 ± 0.005 ^a^
10	Cysteine	0.08 ± 0.001 ^c^	0.09 ± 0.01 ^bc^	0.1 ± 0.01 ^bc^	0.2 ± 0.02 ^a^
11	Tyrosine	0.3 ± 0.021 ^c^	0.3 ± 0.006 ^c^	0.4 ± 0.008 ^b^	0.7 ± 0.014 ^a^
12	Valine	0.4 ± 0.009 ^c^	0.5 ± 0.04 ^c^	0.6 ± 0.05 ^b^	0.9 ± 0.09 ^a^
13	Methionine	0.2 ± 0.01 ^c^	0.2 ± 0.004 ^c^	0.3 ± 0.006 ^b^	0.3 ± 0.006 ^a^
14	Tryptophan	0.1 ± 0.001 ^d^	0.1 ± 0.002 ^c^	0.1 ± 0.003 ^b^	0.2 ± 0.005 ^a^
15	Isoleucine	0.3 ± 0.03 ^c^	0.4 ± 0.03 ^c^	0.5 ± 0.05 ^b^	0.8 ± 0.07 ^a^
16	Leucine	0.6 ± 0.006 ^d^	0.7 ± 0.007 ^c^	0.9 ± 0.01 ^b^	1.5 ± 0.01 ^a^
17	Phenylalanine	0.4 ± 0.01 ^c^	0.5 ± 0.05 ^bc^	0.7 ± 0.07 ^b^	1.2 ± 0.13 ^a^

Values represent means of three replicates ± SD. Within each row, means followed by same letter do not differ significantly at *p ≤* 0.05, derived from Tukey’s HSD.

## Data Availability

Not applicable.
